# Salinity and Salt-Priming Impact on Growth, Photosynthetic Performance, and Nutritional Quality of Edible *Mesembryanthemum crystallinum* L.

**DOI:** 10.3390/plants11030332

**Published:** 2022-01-26

**Authors:** Jie He, Olivia Wei Jin Ng, Lin Qin

**Affiliations:** Natural Sciences and Science Education Academic Group, National Institute of Education, Nanyang Technological University, 1 Nanyang Walk, Singapore 637616, Singapore; oliviang19@gmail.com (O.W.J.N.); lin.qin@nie.edu.sg (L.Q.)

**Keywords:** artificial seawater, halophyte, leaf growth, photosynthesis, phytochemicals, salinity stress, salt priming

## Abstract

*Mesembryanthemum crystallinum* L. is a nutritious edible facultative halophyte. This study aimed to investigate the physiology and quality of *M. crystallinum* L. grown under different salinities and salt-priming conditions. All plants were first grown in 10% artificial seawater (ASW) for 10 days. After that, some plants remained in 10% ASW while the others were transferred to 20%, 30%, 40%, or 50% ASW for another 10 days. Some plants also underwent a salt priming by transferring them gradually from 10% to 100% ASW over a span of 10 days (defined as salt primed). All plants were green and healthy. However, there were reductions in shoot and root productivity, leaf growth, and water content, but also an increase in leaf succulence after transferring plants to higher salinities. The salt-primed plants showed higher photosynthetic light use efficiency with higher chlorophyll concentration compared to other plants. The concentrations of proline, ascorbic acid (ASC), and total phenolic compounds (TPC) increased as percentages of ASW increased. The salt-primed plants switched from C_3_ to crassulacean acid metabolism photosynthesis and accumulated the greatest amounts of proline, ASC, and TPC. In conclusion, higher salinities and salt priming enhance the nutritional quality of *M. crystallinum* L. but compromises productivity.

## 1. Introduction

Native to southern and eastern Africa, *M. crystallinum* L. (common ice plant) is a facultative halophyte, which means saline is not a physical requirement for growth [1]. However, some researchers consider that *M. crystallinum* L. belongs to the group “obligatory” halophytes, which requires saline environments for optimal growth [2]. It has high economical value as it has many important uses. Its leaves and stems can be used as human food [3]. It also has antioxidant and antimicrobial properties that helps to reduce inflammation of the mucous membranes in respiratory and urinary systems [4]. The maintenance of food security, in terms of quantity and quality, is an increasing challenge for Singapore due to limited land. In Singapore, almost 90% of food is imported from an increasingly disrupted world resulting from the COVID-19 pandemic. Furthermore, major food production areas are expected to face reduced water availability and increased drought frequency due to climate change [5]. In March 2019, the Singapore Food Agency has set a goal of achieving “30 by 30”, which is to develop the capability and capacity of our agri-food industry to locally produce 30% of Singapore’s nutritional needs by 2030 [6]. To ensure that such needs could be met, mass-produced halophytes as vegetables, using saline resources, could be one of the strategies to address water issues.

According to Flowers et al. [7], halophytes are plants that are able to complete their lifecycle in a salt concentration of at least 200 mM NaCl under conditions similar to those that might be encountered in the natural environment. Halophytes can tolerate salinity stress as they possess salt responsive genes and proteins to counter the adverse effects of salinity [8,9]. However, tolerance to salinity stress varies among halophyte species [10,11]. Halophytes that are less tolerant to salinity normally reduce their growth in saline environment [12]. Reduced growth under salinity conditions helps the plants to conserve energy for the production of osmolytes, which protect halophytes against hyperosmotic stress [13]. Osmolytes, such as free proline and soluble sugars, defined as having health-promoting benefits, can potentially be used in functional food [14,15]. Soluble sugars and proline could be used by plants in osmotic adjustments [16]. However, it has been reported that halophytes may not be able to survive and thrive under extremely saline conditions [17]. For protection against the oxidative stress caused by salinity, antioxidants such as ascorbic acid (ASC) and phenolic compounds are produced [18,19]. Given that these antioxidants are also beneficial for human health [20], the awareness of a healthier diet and halophytes with high nutritional values may promote additional markets for some halophytic vegetables [21].

Sivritepe et al. [22] reported that seed salt priming is a useful technique to improve the salt tolerance of plants because seeds that have been primed are better able to adjust osmotically. This is because primed seeds have high levels of Na and Cl in their roots as well as high concentrations of soluble sugars and organic acids in their leaves compared to those non-primed seeds [23]. Memory of the first stress (priming) and retrieval of the remembered information upon encounter with the later stress are necessary to improve stress tolerance [23]. Salt-priming treatments have been mainly applied to seeds, and the possible impacts of seed priming on plant growth and development and different physiological processes were monitored during seed germination and seedling stage [24,25,26]. It was reported that NaCl priming improved the germination and seedling growth of canola (*Brassica napus*) [24] and pepper (*Capsicum annuum* L.) [25]. NaCl priming resulted in better cell membrane stability compared to untreated seedlings [24,25]. Farhoudi [26] reported that seed priming improved antioxidant activity and compatible solute content such as sugars and proline in Muskmelon (*Cucumis melo* L.) under salt stress. However, there is very little research on salt priming during the vegetative stage.

In the study with *M. crystallinum* L., we have found that plants grown in 100 mM NaCl had the highest productivity compared to those grown either at higher salinities or in freshwater [27]. In another recent study, we have also reported that *M. crystallinum* L. grown under 500 mM NaCl had decreased leaf growth, biomass accumulation, and leaf water content compared to those grown under lower concentration of NaCl [28]. However, all plants were healthy with similar maximal efficiency of photosystem II (PS II) photochemistry. Plants grown under 500 mM NaCl switched from C_3_ photosynthesis to CAM photosynthesis in order to tolerate salinity stress. CAM is characterized by nocturnal CO_2_ uptake, which increased water-use efficiency when compared to C_3_ plants [29]. Phytochemicals such as proline, total soluble sugar (TSS), ASC and total phenolic compounds (TPC) were significantly higher in plants grown under higher salinity compared to those grown under lower salinity. However, shoot productivity was 10-fold lower in *M. crystallinum* L. grown in 500 mM NaCl compared with those grown in 100 mM NaCl [28]. Therefore, this study aimed to investigate how the changes in the salinity conditions of ASW affect plant growth, water relations, photosynthetic performance, and the nutritional qualities of *M. crystallinum* L. It was also hypnotized that salt priming by transferring *M. crystallinum* L. at active growth stage from low salinity of ASW gradually to higher salinities of ASW would enhance the salt tolerance and improve the nutritional values of this edible halophyte. The findings of this study could be beneficial in the local context if it is feasible to grow *M. crystallinum* L. using seawater in Singapore to ensure food security and sustainability.

## 2. Results

### 2.1. Productivity

**Figure 1:**
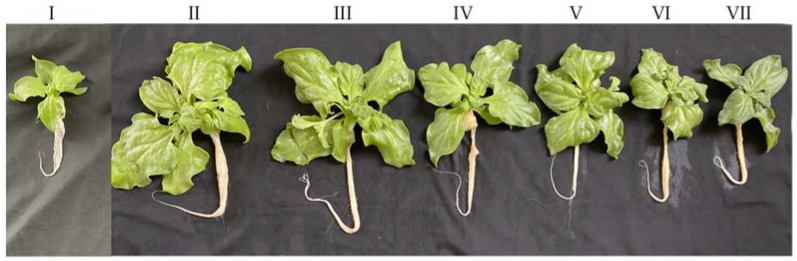
*M. crystallinum* L. grown indoor hydroponically for 10 days (I) and 20 days (II to VII). I: 10% artificial seawater (ASW) for 10 days; II: 10% ASW for 20 days; III: 10% ASW, 10 days ⟹ 20% ASW, 10 days; IV: 10% ASW, 10 days ⟹ 30% ASW, 10 days; V: 10% ASW, 10 days ⟹ 40% ASW, 10 days; VI: 10% ASW, 10 days ⟹ 50% ASW, 10 days; VII: 10% ASW, 10 days ⟹ 20% ASW, 2 days ⟹ 40% ASW, 2 days ⟹ 60% ASW, 2 days ⟹ 80% ASW, 2 days ⟹ 100% ASW, 2 days (salt-primed).

**Figure 2:**
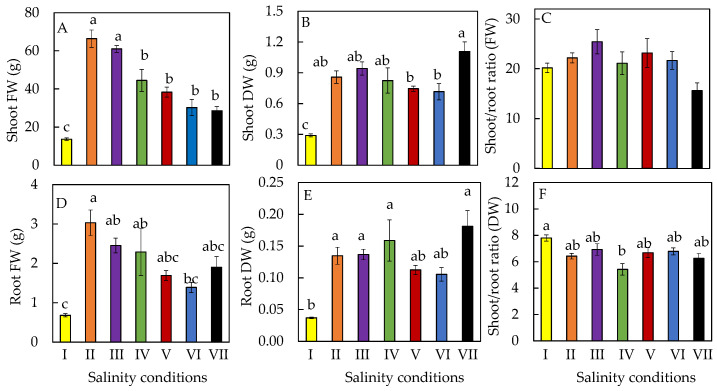
Shoot FW and DW (**A**,**B**), root FW and DW (**D**,**E**), and shoot/root ratio FW and DW (**C**,**F**) of *M. crystallinum* L. grown under different salinity conditions for 10 or 20 days. Values are means (±S.E) of four replicates from four different plants, and different letters indicate significant differences at *p* < 0.05. When letters are absent, there were no significant differences between the treatments. Refer to Figure 1 legend for different salinity conditions of I, II, III, IV, V, VI, and VII.

Figure 1 shows that all *M. crystallinum* L. grew healthily after transferring them to different salinities. Compared to the 10-day-old plants grown in 10% ASW (salinity condition I), all other plants transferred to higher percentages of ASW (salinity conditions II to VII) continued to grow bigger (Figure 1) as supported by their higher shoot and root FW and DW after another 10 days of growth (Figure 2A,B,D,E). Plants that were transferred from 10% ASW to 20% ASW for 10 days had similar shoot FW as those that remained growing in 10% ASW for another 10 days. However, after changing the salinities from 10% ASW to higher percentages of ASW (salinity conditions III to VI), shoot and root productivity significantly declined. Furthermore, plants that were gradually transferred from 10% ASW to 100% ASW (condition VII, defined as salt-primed plants) had the lower shoot FW (Figure 2A) but the highest shoot and root DW (Figure 2B,E). It was noticed that the root FW of *M. crystallinum* L. grown in 10% ASW for 10 days (condition I, Figure 2D) was less than half of those grown in higher concentrations of ASW (conditions V, VI, VII) for 20 days. However, due to the large standard errors, there were no significant differences in root FW detected among these plants. A similar situation was also seen in root DW (Figure 2E). There were generally no significant differences in the shoot/root ratio (FW) and shoot/root ratio (DW) of *M. crystallinum* L. after changing to higher salinities (Figure 2C,F).

### 2.2. Leaf Growth

**Figure 3:**
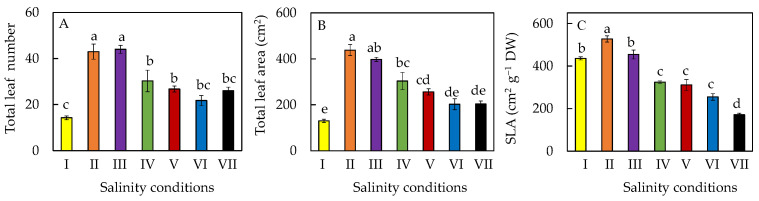
Total leaf number (**A**), total leaf area (**B**), and SLA (**C**) of *M. crystallinum* L. grown under different salinity conditions for 10 or 20 days. Values are means (±S.E) of four replicates from four different plants, and different letters indicate significant differences at *p* < 0.05. Refer to Figure 1 legend for different salinity conditions of I, II, III, IV, V, VI, and VII.

Generally, total leaf number and total leaf area were smaller after the plants were transferred from 10% ASW to higher salinity conditions in ASW ≥30% (Figure 3A,B). However, total leaf number and total leaf area of plants that were transferred from 10% ASW to 20% ASW were statistically similar to the plants that remained growing in 10% ASW. Plants grown in 10% ASW for 20 days (salinity condition II) had the highest specific leaf area (SLA), while salt-primed plants (salinity condition VII) had a significantly lower SLA compared to those of the rest of the plants (Figure 3C).

### 2.3. Leaf Water Status

**Figure 4:**
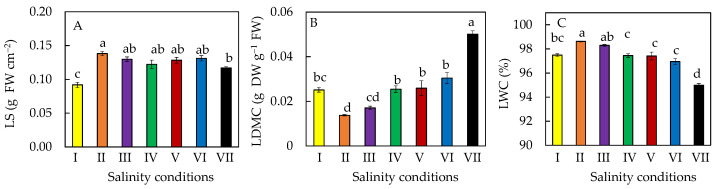
LS (**A**), LDMC (**B**), and LWC (**C**) of *M. crystallinum* L. grown under different salinity conditions for 10 or 20 days. Values are means (±S.E) of four replicates from four different plants, and different letters indicate significant differences at *p* < 0.05. Refer to Figure 1 legend for different salinity conditions of I, II, III, IV, V, VI, and VII.

*M. crystallinum* L. plants grown under different salinity conditions for 20 days (conditions II to VII) had significantly higher leaf succulence (LS) compared to those grown in 10% ASW (condition I) for only 10 days (Figure 4A). However, salt-primed plants (condition VII) had a significantly lower LS compared to those grown in 20% ASW (conditions II) at the same growth stage (Figure 4A). After transferring to different percentages of ASW, leaf dry matter content (LDMC) accumulation increased with increasing salinities (Figure 4B). However, this was the opposite for leaf water content (LWC), where plants that were gradually transferred from 10% ASW to 100% ASW (salt-primed plants) had the lowest LWC (Figure 4C). 

### 2.4. Chlorophyll (Chl) Fluorescence F_v_/F_m_ (Variable/Maximal Fluorescence) Ratio and CAM (Crassulacean Acid Metabolism) Acidity

**Figure 5:**
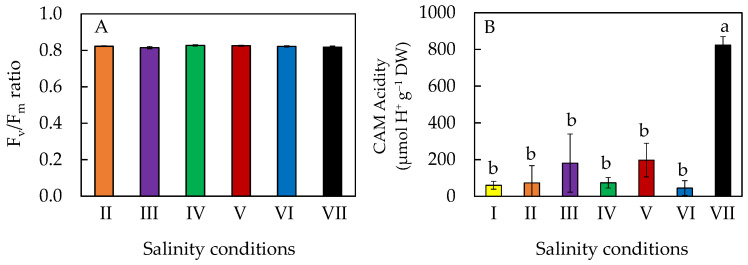
F_v_/F_m_ ratio (**A**) and CAM acidity (**B**) of *M. crystallinum* L. grown under different salinity conditions for 10 or 20 days. Values are means (±S.E) of four replicates from four different plants, and different letters indicate significant differences at *p* < 0.05. When letters are absent, there were no significant differences between the treatments. Refer to Figure 1 legend for different salinity conditions of I, II, III, IV, V, VI, and VII.

All *M. crystallinum* L. had similar values of F_v_/F_m_ ratio, which were close to 0.8, indicating that the plants were healthy (Figure 5A). CAM acidity was significantly higher in salt-primed plants (Figure 5B), which was about four to eight times higher compared to those grown in the other salinity conditions. *M. crystallinum* L. grown in conditions III and V had CAM acidity more than two times higher than those grown in conditions I, II, IV, and VI. However, due to the large standard errors, there were so significant differences in CAM acidity detected among these plants (Figure 5B). 

### 2.5. Photosynthetic Pigments

**Figure 6:**
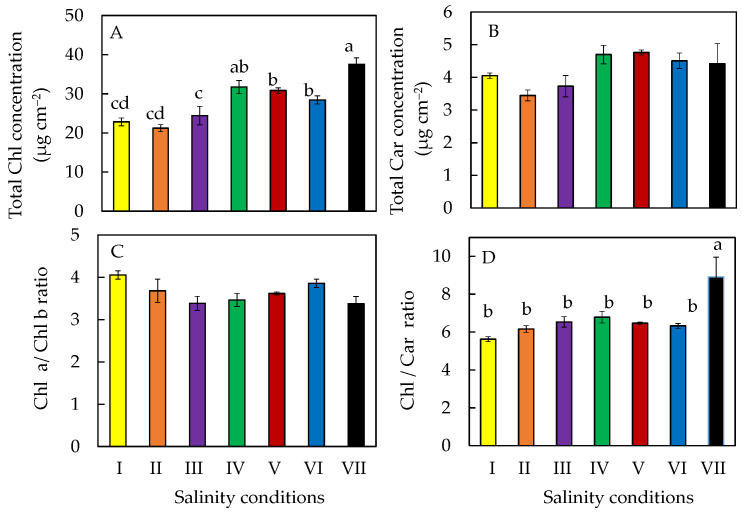
Total Chl concentration (**A**), total Car concentration (**B**), Chl a/b ratio (**C**), and Chl/Car ratio (**D**) of *M. crystallinum* L. grown under different salinity conditions for 10 or 20 days. Values are means (±S.E) of four replicates from four different plants, and different letters indicate significant differences at *p* < 0.05. When letters are absent, there were no significant differences between the treatments. Refer to Figure 1 legend for different salinity conditions of I, II, III, IV, V, VI, and VII.

Total Chl concentration and Chl/Car ratio were significantly highest in salt-primed plants (Figure 6A,D). No significant differences were observed for the total Car concentration (Figure 6B) and Chl a/Chl b ratio (Figure 6C) after transferring the plants to different salinity conditions compared to those of salt-primed plants.

### 2.6. Electron Transport Rate (ETR), Effective Quantum Yield of PS II (*Δ*F/F_m_′) and Non-Photochemical Quenching (NPQ)

**Figure 7:**
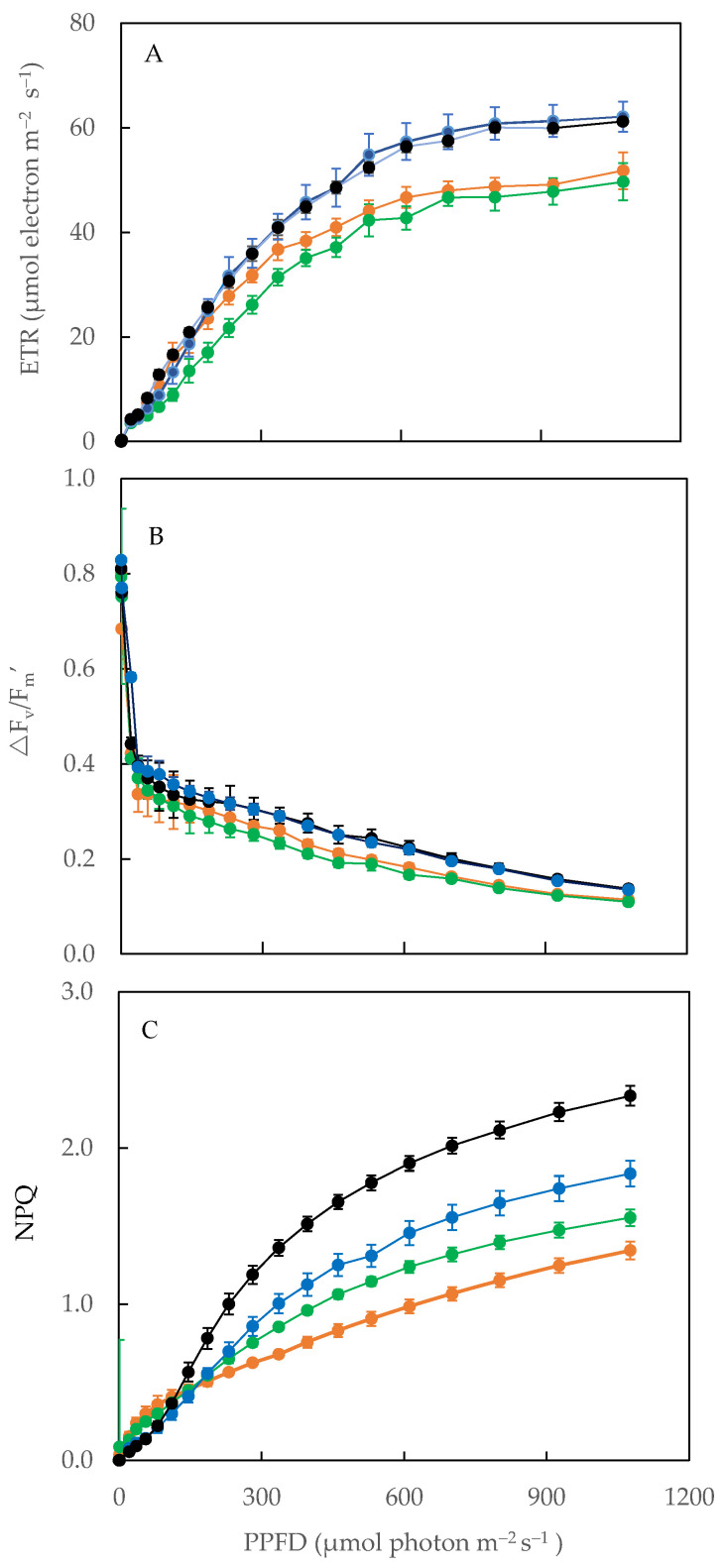
Light response curves of ETR (**A**), ΔF/F_m_’ (**B**), and NPQ (**C**) of *M. crystallinum* L. grown under different salinity conditions for 10 or 20 days. Values are means (±S.E.) with replicates of four from four different plants. ●, 10% ASW for 20 days; ●, 10% ASW, 10 days ⟹ 30% ASW, 10 days; ●, 10% ASW, 10 days ⟹ 50% ASW, 10 days; ●, 10% ASW, 10 days ⟹ 20% ASW, 2 days ⟹ 40% ASW, 2 days ⟹ 60% ASW, 2 days ⟹ 80% ASW, 2 days ⟹ 100% ASW, 2 days (salt-primed plants).

The light responses curves of ETR, ΔF/F_m_′ and NPQ were measured for all *M. crystallinum* L. after transferring them to different salinity conditions. For better clarity, Figure 7 only shows the light response curves of plants grown under four salinity conditions. For all plants, the light response curves of ETR and NPQ showed an upward trend with increasing photosynthetic photon flux density (PPFD) (Figure 7A,C) while the curve of ΔF/F_m_′ showed an opposite trend (Figure 7B). When comparisons were made between different plants that were transferred to different salinity conditions, *M. crystallinum* L. transferred from 10% to 50% ASW and salt-primed plants had higher values of ETR, NPQ, and ΔF/F_m_′ compared to those of the other two conditions (Figure 7A,C) when measured under the same PPFDs.

**Figure 8:**
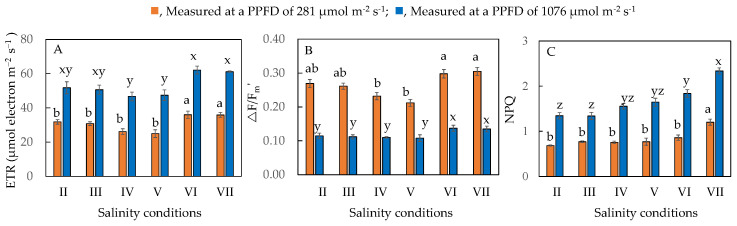
ETR (**A**), ΔF/F_m_′ (**B**), and NPQ (**C**), measured at a PPFD of 281 µmol m^−2^ s^−1^, which is near the growth irradiance, and at a maximal PPFD of 1076 µmol m^−2^ s^−1,^ of *M. crystallinum* L. grown under different salinity conditions for 20 days. Values are means (±S.E) of four replicates from four different plants, and different letters indicate significant differences at *p* < 0.05. Refer to Figure 1 legend for different salinity conditions of II, III, IV, V, VI, and VII.

Figure 8 shows the values of ETR, ΔF/F_m_′ and NPQ, measured under a PPFD of 281 µmol m^−2^ s^−1^, which was near the growth irradiance and under a maximal PPFD of 1076 µmol m^−2^ s^−1^ for *M. crystallinum* L. grown under different salinities for 20 days. Under both PPFDs, ETR, NPQ, and ΔF/F_m_′ were significantly the highest in salt-primed *M. crystallinum* L. compared to all other plants. Except for *M. crystallinum* L. transferred from 10% to 50% ASW, which had similar ETR and ΔF/F_m_′ to those salt-primed plants, all other plants grown under a lower percentage of ASW had similar lower values of ETR, NPQ, and ΔF/F_m_′. 

### 2.7. Accumulation of Phytochemicals 

**Figure 9:**
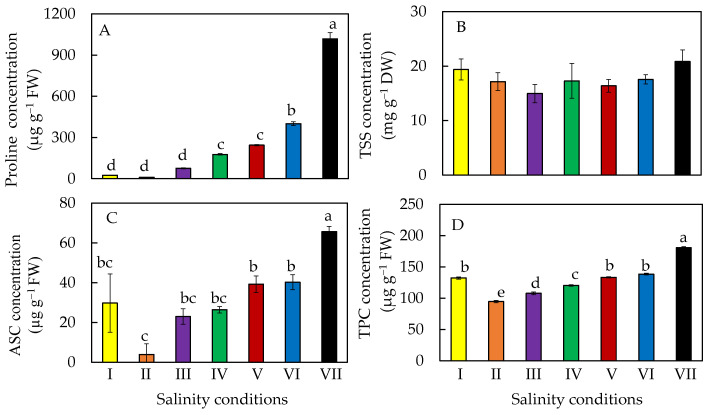
Proline (**A**), TSS (**B**), ASC (**C**), and TPC (**D**) concentrations of *M. crystallinum* L. grown under different salinity conditions for 10 or 20 days. Values are means (±S.E) of four replicates from four different plants, and different letters indicate significant differences at *p* < 0.05. When letters are absent, there were no significant differences between the treatments. Refer to Figure 1 legend for different salinity conditions of I, II, III, IV, V, VI, and VII.

Proline concentration increased after salinity conditions were changed from 10% ASW to ≥20% ASW (Figure 9A). The salt-primed plants had the highest proline concentration followed by those transferred from 10% ASW to 50% ASW, and then by 40% ASW and 30% ASW. Statistically, there were no significant differences in proline concentration between plants grown in 10% ASW for 10 and 20 days and those transferred from 10% ASW to 20% ASW (Figure 9A), which were significantly lower than those grown under higher salinity conditions. However, all plants had similar amounts of TSS (Figure 9B). Similar increasing trends were observed for the concentrations of ASC (Figure 9C) and TPC (Figure 9D). The salt-primed plants had the highest ASC and TPC concentrations compared to those of plants grown in the other salinity conditions (Figure 9C,D). 

## 3. Discussion

In this study, all *M. crystallinum* L. plants were first grown in 10% ASW for 10 days and then transferred to different salinities with higher percentages of ASW for another 10 days. All plants continued to grow after salinity conditions were changed (Figure 1). *M. crystallinum* L. grown in 10% ASW for the whole growth cycle of 20 days and those first grown in 10% ASW for the first 10 days followed by another 10 days in 20% ASW exhibited the best growth. This was supported by their highest shoot and root FW (Figure 2A,D). The high shoot FW is mainly due to the fact that plants grown in these two conditions had the greatest total leaf number and largest total leaf area compared to those grown in the other salinity conditions (Figure 3A,B). It was reported that, for plants subjected to salt stress, a general decrease in plant FW is often observed due to a reduction in the number of leaves or leaf abscissions [30]. The reduction in the leaf area for plants grown at higher salinity conditions may be an avoidance mechanism used by the plant to minimize water loss during transpiration [30,31,32]. However, the shoot and root DW were the highest in plants gradually transferred from 10% ASW to 100% ASW (Figure 2B,D) because they had the lowest LWC amongst the other plants (Figure 4C). Similar results were also obtained where spinach was grown in different concentrations of salt [31]. It was reported that a linear increase in DW and a linear decrease in water content with increasing salinity stress could be due to the Na^+^ accrual [33]. Furthermore, LDMC was the highest (Figure 4B) in salt-primed *M. crystallinum* L., accounting for the lowest SLA (Figure 3C) and thicker leaves. A similar trend was also observed in spinach *Spinacia oleracea* (L.), where the increased thickness may be associated with an increase in cell size [33]. Generally, no significant differences were observed in the shoot/root ratio (FW or DW) of *M. crystallinum* L. after changing to higher salinities (Figure 2C,F). These results indicate that the changes in salinity do not alter the resource allocation in *M. crystallinum* L., thus, photoassimilate partitioning between root and shoot remains constant. However, contradictory results were reported by another team, who found that the shoot/root ratio increased when *M. crystallinum* L. were grown under salinity [34]. An increased shoot/root ratio implies that the carbohydrate demands of CAM in the photosynthetic shoot take priority over root growth under water-limited conditions [34]. There were no significant differences in LS after the *M. crystallinum* L. plants were transferred from 10% ASW to different higher percentages of ASW (Figure 4A). These results imply that the salt-primed *M. crystallinum* L. in this study switched to CAM under high salinity conditions (Figure 5B) and increased water-use efficiency and thus maintained LS as high as those *M. crystallinum* L. that perform C_3_ photosynthesis under non-stressful lower salinity conditions [29]. Similar results were also obtained in our previous work, which reported that salinity did not affect the LS of *M. crystallinum* L. [27]. However, LS was significantly higher for all the plants grown in higher salinities for 20 days compared to those grown in 10% ASW for only 10 days. Plants may increase LS to dilute salts [27]. It was found that the LS was significantly lower in salt-primed *M. crystallinum* L. compared to those grown in 10% ASW for 20 days, which could be due to its greater decrease in LWC. Lower LS and LWC in salt-primed *M. crystallinum* L. grown with 100% ASW could be attributed to the stunted root architecture, which might have limited water uptake [28].

F_v_/F_m_ ratio measured from dark-adapted leaves provides important information on the maximal efficiency of PS II photochemistry [35]. F_v_/F_m_ ratios of all plants were close to 0.8 (Figure 5A), indicating that all were healthy and there was no evidence of damage to PS II [36]. These results concur with our previous findings that *M. crystallinum* L. grown in 100 mM, 250 mM, and 500 mM NaCl had its F_v_/F_m_ ratio close to 0.8 [28]. However, significant reductions in F_v_/F_m_ ratios were observed in plants such as *Arabidopsis thaliana* [37] and lettuce (*Lactuca sativa*) [38] grown under higher NaCl concentrations. Measured under different actinic light, salt-primed *M. crystallinum* L. grown in 100% ASW generally showed a higher ETR, ΔF/F_m_’ and NPQ compared to those of plants grown at lower salinities (Figure 7 and Figure 8). Plants respond to salt stress by increasing NPQ as a mechanism to safely dissipate excess energy and minimize reactive oxygen species (ROS) generation [30]. It was also reported that photochemical quenching (qP) and ETR decreased under salt stress [30], which contradicts the results of this study. It was likely that *M. crystallinum* L. had undergone salt priming over 10 days from 10% ASW to 100% ASW, increased its light use efficiency, and thus had a higher ETR and effective quantum yield, ΔF/F_m_’. A study carried out by Wang et al. [39] found that plants that underwent drought priming could better cope with drought stress as they accumulated osmolytes to maintain high turgor pressure and have higher antioxidant enzyme activity to prevent cell membrane damage in leaves and roots. For *M. crystallinum* L. that were salt primed, they could have developed similar adaptive mechanisms through the accumulation of antioxidants (Figure 9, to be discussed next) that helped them to continue to thrive in high salt concentrations, thus accounting for the increase in ETR and ΔF/F_m_′ under higher salinity. 

It was reported that total Chl might reduce under salinity stress, resulting from increased Chl degradation and reduced Chl synthesis [40]. However, in this study, total Chl concentration was the highest in salt-primed plants grown in 100% ASW (Figure 6A). It has also been reported that salt tolerant species respond to salt stress by maintaining or increasing their Chl concentration as a mechanism to protect the photosynthesis process [30]. Furthermore, salt-primed *M. crystallinum* L. was also observed to have the smallest total leaf area and SLA amongst the other plants (Figure 3B,C). These findings suggest that Chl was being concentrated in the small but thicker leaves, thus accounting for the higher total Chl concentration. However, all plants had similar Chl a/b ratios (Figure 6C). No significant differences were observed in total Car concentration between the different salinity treatments (Figure 6B). This could explain why there was no significant differences in Chl/Car ratios except for the salt-primed plants, which had a significantly higher Chl/Car ratio compared to all other plants (Figure 6D). 

CAM acidity of salt-primed *M. crystallinum* L., which were gradually transferred from 10% ASW to 100% ASW was 4 to 8-fold higher than those grown in the other salinity conditions (Figure 5B). This result agrees with the recent study by Guan et al. [41], which indicates that when *M. crystallinum* L. is under high salt stress, it has an adaptive mechanism of switching its photosynthetic mode from C_3_ photosynthesis to CAM to enhance water use efficiency. When plants use CAM photosynthesis, photorespiration is reduced as they only open their stomata in the night to prevent water loss. It was suggested that NPQ can be used to estimate the degree of CAM induction [19,35]. For sat-primed plants, CAM acidity was significantly higher with higher values of NPQ (Figure 5B and Figure 7C). Because CAM is an energetically expensive process, the induction of CAM for salt-primed *M. crystallinum* L. could potentially account for its low shoot biomass accumulation (Figure 2A). 

Hsouna et al. [42] have recently suggested that plants accumulate osmolytes such as proline as one of the strategies to avoid the consequences of stress caused by high salinity. In this study, proline concentration in *M. crystallinum* L. increased after transferring plants to higher percentages of ASW (Figure 9A). He et al. [28] also reported that *M. crystallinum* L. grown in 250 mM and 500 mM of NaCl had a higher proline accumulation compared to the plants grown in 100 mM of NaCl. Salt-primed *M. crystallinum* grown in 100% ASW had the highest proline concentration (Figure 9A), suggesting that salt priming could further enhance proline accumulation. Halophytes are able to tolerate salt and maintain their growth through osmotic adjustment, which is achieved by accumulating compatible solutes such as proline or TSS [43]. However, all plants had similar TSS concentrations regardless of the salinity under which *M. crystallinum* L. were grown (Figure 9B). Salt stress is known to enhance the production of ROS in plants, which can cause oxidative damage to lipids, proteins, and nucleic acids [44]. It is well known that ASC alleviates the oxidative stress in plants subjected to salinity stress by deactivating the ROS. As *M. crystallinum* L. was gradually transferred from 10% ASW to 100% ASW, the greatest amount of ASC was accumulated (Figure 9C). This result suggests that salt priming increases plant protection against oxidative stress. Cayuela et al. [23] reported that phenolic compounds have antioxidant properties and can confer various physiological responses to stresses in plants. This further supports the result of this study with the greatest amount of TPC found in salt-primed *M. crystallinum* L. (Figure 9D). 

In conclusion, grown in higher salinity or under salt-priming conditions, *M. crystallinum* L. exhibited a high level of salt tolerance, with survival up to 100% ASW. At lower salinity conditions, *M. crystallinum* L. were more productive but had lower nutrition qualities. There were increases in the concentrations of phytochemicals such as proline, ASC, and TPC with increasing salinities. High accumulations of proline, ASC, and TPC in the shoot, which is mainly made up of leaves, increased the protection of *M. crystallinum* L. again salinity stress and enhanced the nutritional quality of edible *M. crystallinum* L. However, the productivity of *M. crystallinum* L. was compromised at higher salinity or salt-priming conditions. Although salt-primed *M. crystallinum* L. grown in 100% ASW had the lowest shoot productivity in this study, they had a greater yield compared to those grown in 500 mM NaCl (salinity similar to 100% ASW), reported by our previous study [27]. The results of this study provide *M. crystallinum* L. growers a better understanding of the optimal conditions under which plants should grow to achieve specific results, such as high nutritional quality, without substantial yield reduction.

## 4. Materials and Methods

### 4.1. Plant Materials and Experimental Design

Seeds of *M. crystallinum* L. were germinated on moist filter paper in petri dishes. The seedlings were then inserted into polyurethane cubes. They were incubated under a PPFD of 100 µmol m^−2^ s^−1^ provided by high-pressure sodium lamps for four weeks before transplanting into an indoor hydroponic system. They were grown under a red/blue (R/B) LED ratio of 2.2 (WR-16W, Beijing Lighting Valley Technology Co., Ltd., Beijing, China) and exposed to an average PPFD of 240 µmol m^−2^ s^−1^, for a 12 h photoperiod from 08.00 to 20.00 daily. All the seedlings were grown in 10% ASW with full strength nutrient solution (2.2 ± 0.2 mS cm^−1^ conductivity and pH 6.0 ± 0.2) for 10 days before they were transferred to different salinity conditions in 20%, 30%, 40%, and 50% ASW, with full strength nutrient solution for another 10 days. In this study, 100% ASW with the salinity of 33 ppt was prepared by dissolving 36 g of Red Sea Salt^®^ (Red Sea Fish Pharm Ltd., Eilat, Israel) in 1 L water. Different salinity conditions (10%, 20%, 30%, 40%, and 50% ASW) were obtained by dissolving 28.8 g, 57.6 g, 86.4 g, 115.2 g, and 144 g of Red Sea Salt^®^ in 8 L full strength nutrient solution. There was also an additional salt-priming treatment, that was, some of the seedlings were transferred to 20% ASW for two days before being transferred to increasingly higher salinities of 40%, 60%, 80% and 100% ASW for two days of each transfer (defined as salt-primed). In the salt-primed treatment, the addition of Red Sea Salt^®^ was added every two days to reach increasingly higher salinities. A total of two rounds of harvesting were conducted—once after the seedlings were grown in 10% ASW for 10 days and once after the seedlings had been transferred to different salinities for another 10 days. The room temperature and relative humidity were 24.5 °C/23 °C and 60%/80% (day/night), respectively.

### 4.2. Productivity, Leaf Growth and Leaf Water Status

As shown in Figure 1, all *M. crystallinum* L. plants used for this study were stemless with rosette leaves. The stemless *M. crystallinum* L. plants have no visible stem above ground and are composed mainly of leaves. Shoots were used for the productivity of aerial parts, where leaves were used when measuring their number, area, and water status. For each harvest, four plants from each treatment were harvested. The total leaf number was recorded before the polyurethane cubes were removed to separate shoot and root for their FW measurements. The youngest fully expanded leaves were also weighed separately. The total leaf area of each plant was determined using a leaf area meter (WinDIAS3 Image Analysis system). To obtain DW, the shoots, roots, and youngest fully expanded leaves were dried separately in an oven at 80 °C for at least four days. SLA was calculated using L_A_/L_DW_, where L_A_ = leaf area (cm^2^) and L_DW_ = leaf dry weight (g) [45]. LS was calculated using L_FW_/L_A_, where L_FW_ = leaf fresh weight (g) [46]. LDMC was determined by L_DW_/L_FW_ [47], while LWC was determined by (L_FW_ − L_DW_)/L_FW_. 

### 4.3. Measurement of Chl Fluorescence F_v_/F_m_ Ratio

The maximal efficiency of PS II photochemistry was measured by the fluorescence F_v_/F_m_ ratio from the dark-adapted leaves (15 min in darkness) using a Plant Efficiency Analyser (Hansatech Instruments Ltd., England) during the mid-photoperiod [48]. The initial fluorescence, F_o_, was first recorded. F_m_, maximum fluorescence, was assessed by 0.8 s of saturated pulse (>6000 μmquol∙m^−2^ s^−1^, which completely reduces all primary acceptors for PS II). Variable fluorescence, F_v_, was calculated as F_v_ = F_m_ − F_o_. 

### 4.4. Determination of CAM Acidity 

Leaf discs were punched out of each plant sample before and at the end of the photoperiod [49]. The leaf discs were transferred to microtiter plate wells containing 1 mL of Milli-Q water before being heated in a 95 °C water bath for 15 min. The extracts were subsequently titrated against 0.01 M sodium hydroxide solution, NaOH (aq), and three drops of phenolphthalein was used as an indicator until the end point was reached. A final volume of NaOH was used to reach the end-point and was used to calculate CAM acidity as μmol H^+^ g^−1^ DW by first using the formula: [0.01 × volume of NaOH (aq)]/DW, followed by obtaining the difference in this calculated value immediately before and at the end of the photoperiod.

### 4.5. Determinations of Chl and Car Concentrations

Fresh leaf discs of 0.05 g (converted to area, cm^2^ for calculation) were obtained from each plant sample and soaked in 5 mL of N, N-dimethylformamide (Sigma chemical co., Sigma-Aldrich, MO, USA) in the dark for 48 h at 4 °C. A spectrophotometer (UV-2550 Shimadzu, Japan) was used to measure the absorption of Chl a, Chl b, and carotenoids at 647 nm, 664 nm, and 480 nm, respectively. Chl and Car concentrations were calculated according to Welburn [50]:

Chl a (µg cm^−2^) = (11.65 × A_664_ – 2.69 × A_647_) × vol. × 1/leaf area (cm^2^)

Chl b (µg cm^−2^) = (20.81 × A_647_ – 4.53 × A_664_) × vol. × 1/leaf area (cm^2^)

Total Chl (µg cm^−2^) = Chl a + Chl b

Car (µg cm^−2^) = (1000 × A_480_ – 0.89 × Ca’ – 52.02 × Cb’)/245 × vol. × 1/leaf area (cm^2^)

Ca’ = 11.65 × A_664_ – 2.69 × A_647_, Cb’ = 20.81 × A_647_ – 4.53 × A_664_

### 4.6. Measurements of ETR, *Δ*F/F_m_′ and NPQ

The youngest fully expanded leaf from each plant sample was pre-darkened for 15 min prior to measurements. The ETR, ΔF/F_m_′, and NPQ were determined using an Imaging PAM Chl Fluorometer (Walz, Effeltrich, Germany). The images of fluorescence emission were digitized within the camera and transmitted via a Firewire interface (400 megabits/s) (Firewire-1394, Austin, TX, USA) to a personal computer for storage and analysis. Measurements and calculations of ΔF/F_m_′, ETR, and NPQ were determined according to He et al. [48] using the following equation: ΔF/F_m_^′^ = [(F_m_′ − F)/F_m_′)] and ETR = (PPFD × ΔF/F_m_^′^ × 0.5 × 0.84). The number of 0.5 represents a supposition that the excitations are equally distributed between PS II and PS I. Correction factor 0.84 takes into account that only a fraction of incident light is really absorbed by photosynthesis. NPQ was defined as: NPQ = (F_m_ − F_m_′)/F_m_′.

### 4.7. Determination of Proline

Fresh leaves of 1 g from each plant sample were ground with 12 mL of 3% sulfosalicylic acid and centrifuged at 9000 rpm for 10 min at 4 °C; 1 mL of the supernatant was then added to 1 mL of acid-ninhydrin and 1 mL of acetic acid. The mixture was then heated for 1 h in a 95 °C water bath. The reaction was stopped in an ice bath. The reaction mixture was then extracted with 2 mL of toluene by vortexing for 30 s and leaving the mixture to stand. A spectrophotometer (UV-2550 Shimadzu, Kyoto, Japan) was used to measure the absorbance at 520 nm, according to Bates et al. [51].

### 4.8. Determination of TSS 

Dried shoot of 0.01 g from each treatment were added to 4 mL of 80% ethanol and heated in a water bath at 65 °C for 30 min. The homogenate was centrifuged at 4000 rpm for 5 min and the supernatant was collected. The pellet was resuspended in an additional 2 mL of 80% ethanol, and the process was repeated twice. The supernatant was topped up to 10 mL with 80% ethanol; 0.5 mL of the supernatant was then added to 0.5 mL of 5% phenol and 2.5 mL of concentrated sulfuric acid. A spectrophotometer (UV-2550 Shimadzu, Japan) was used to measure the absorbance at 490 nm, according to Dubois et al. [52].

### 4.9. Determination of ASC

Fresh leaves of 1 g from each plant sample were extracted in 5 mL of ice-cold 2% (*w*/*v*) metaphosphoric acid. The homogenate was centrifuged at 4 °C and at 9000 rpm for 40 min; 0.3 mL of the supernatant was then added to 0.2 mL of 45% (*w*/*v*) K_2_HPO_4_ and 0.1 mL of 0.1% (*w*/*v*) homocysteine. The mixture was incubated for 15 min at 25 °C before adding 1 mL of 2M citrate–phosphate buffer (pH 2.3) and 1 mL of 0.003% (*w*/*v*) DCPIP. A spectrophotometer (UV-2550 Shimadzu, Japan) was used to measure the absorbance at 524 nm, according to Leipner et al. [53].

### 4.10. Determination of TPC

Fresh leaves of 1 g from each plant were added to 10 mL of 80% methanol. The extracts were shaken for 30 min at 200 rpm and centrifuged for 20 min at 3500 rpm; 0.5 mL of the supernatant was then diluted with 0.5 mL of diluted Folin–Ciocalteau reagent and 1 mL of 7.5% Na_2_CO_3_. After 20 min, a spectrophotometer (UV-2550 Shimadzu, Japan) was used to measure the absorbance at 765 nm, according to Ragee et al. [54]. 

### 4.11. Statistical Analysis

One-way analysis of variance (ANOVA) was performed to determine if there were any statistically significant differences between the treatment groups. When *p*-value < 0.05, post hoc Tukey’s test was used to discriminate the means among the levels of the corresponding factor. Statistical analysis was performed using Minitab. 

## Data Availability

The data supporting the finding of this manuscript are available on reasonable request to the corresponding author.

## References

[1] Adams P., Nelson D.E., Yamada S., Chmara W., Jensen R.G., Bohnert H.J., Griffiths H. (1998). Growth and development of *Mesembryanthemum crystallinum*. New Phytol..

[2] Loconsole D., Murillo-Amador B., Cristiano G., De Lucia B. (2019). Halophyte common ice plants: A future solution to arable land salinization. Sustainability.

[3] Abd El-Gawad A.M., Shehata H.S. (2014). Ecology and development of *Mesembryanthemum crystallinum* L. in the Deltaic Mediterranean coast of Egypt. Egy. J. Basic Appl. Sci..

[4] Ibtissem B., Abdelly C., Sfar S. (2012). Antioxidant and antibacterial properties of *Mesembryanthemum crystallinum* and *Carpobrotus edulis* extracts. Adv. Chem. Eng. Sci..

[5] Iglesias A., Garrote L. (2015). Adaptation strategies for agricultural water management under climate change in Europe. Agric. Water Manag..

[6] Ai-Lien C. (2019). Singapore sets 30% Goal for Home-Grown food by 2030. The Straits Times, Singapore Press Holdings, Singapore. https://www.straitstimes.com/singapore/spore-sets-30-goal-for-home-grown-food-by-2030.

[7] Flowers T.J., Hajibagheri M.A., Clipson N.J.W. (1986). Halophytes. Q. Rev. Biol..

[8] Askari H., Edqvist J., Hajheidari M., Kafi M., Salekdeh G.H. (2006). Effects of salinity levels on proteome of *Suaeda aegyptiaca* leaves. Proteomics.

[9] Yu J., Chen S., Zhao Q., Wang T., Yang C., Diaz C., Sun G., Dai S. (2011). Physiological and proteomic analysis of salinity tolerance in *Puccinellia tenuiflora*. J. Proteome Res..

[10] Flowers T.J., Colmer T.D. (2008). Salinity tolerance in halophytes. New Phytol..

[11] Kumari A., Das P., Parida A.K., Agarwal P.K. (2015). Proteomics, metabolomics, and ionomics perspectives of salinity tolerance in halophytes. Front. Plant Sci..

[12] Zhu J.K. (2001). Plant salt tolerance. Trends Plant Sci..

[13] Slama I., Abdelly C., Bouchereau A., Flower T., Savouré A. (2015). Diversity, distribution and roles of osmoprotective compounds accumulated in halophytes under abiotic stress. Ann. Bot..

[14] Stuchlík M., Žák S. (2002). Vegetable lipids as components of functional foods. Biomed. Pap.-Palacky Univ. Olomouc.

[15] Buhmann A., Papenbrock J. (2013). An economic point of view of secondary compounds in halophytes. Func. Plant Biol..

[16] Flowers T.J. (2004). Improving crop salt tolerance. J. Exp. Bot..

[17] Cheeseman J.M. (2015). The evolution of halophytes, glycophytes and crops, and its implications for food security under saline conditions. New Phytol..

[18] Abd Elgawad H., Zinta G., Hegab M.M., Pandey R., Asard H., Abuelsoud W. (2016). High salinity induces different oxidative stress and antioxidant responses in maize seedlings organs. Front. Plant Sci..

[19] He J., You X., Qin L. (2021). High salinity reduces plant growth and photosynthetic performance but enhances certain nutritional quality of C_4_ Halophyte *Portulaca oleracea* L. grown hydroponically under LED lighting. Front. Plant Sci..

[20] Crozier A., Jaganath I.B., Clifford M.N. (2009). Dietary phenolics: Chemistry, bioavailability and effects on health. Nat. Prod. Rep..

[21] Panta S., Flowers T., Lane P., Doyle R., Haros G., Shabala S. (2014). Halophyte agriculture: Success stories. Environ. Exp. Bot..

[22] Sivritepe N., Sivritepe H.O., Eris A. (2003). The effects of NaCl priming on salt tolerance in melon seedlings grown under saline conditions. Sci. Hortic..

[23] Cayuela E., Perez-Alfocea F., Caro M., Bolarin M.C. (1996). Priming of seeds with NaCl induces physiological changes in tomato plants grown under salt stress. Physiol. Plant..

[24] Farhoudi R., Sharifzadeh F., Poustini K., Makkizadeh M.T., Kochakpor M. (2007). The effects of NaCl priming on salt tolerance in canola (*Brassica napus*) seedlings grown under saline conditions. Seed Sci. Technol..

[25] Khan H.A., Ayub C.M., Pervez M.A., Bilal R.M., Shahid M.A., Ziaf K. (2009). Effect of seed priming with NaCl on salinity tolerance of hot pepper (*Capsicum annuum* L.) at seedling stage. Soil Environ..

[26] Farhoudi1 R., Saeedipour S., Mohammadreza D. (2011). The effect of NaCl seed priming on salt tolerance, antioxidant enzyme activity, proline and carbohydrate accumulation of Muskmelon (*Cucumis melo* L.) under saline condition. Afr. J. Agric. Res..

[27] He J., Qin L. (2020). Productivity and photosynthetic characteristics of the facultative halophyte *Mesembryanthemum crystallinum* grown indoors with LED lighting under different salinities. Acta. Hortic..

[28] He J., Koh D.J.Q., Qin L. (2021). LED spectral quality and NaCl salinity interact to affect growth, photosynthesis and phytochemical production of *Mesembryanthemum crystallinum*. Funct. Plant Biol..

[29] Winter K., Holtum J.A.M. (2014). Facultative crassulacean acid metabolism (CAM) plants: Powerful tools for unravelling the functional elements of CAM photosynthesis. J. Exp. Bot..

[30] Acosta-Motos J., Ortuño M., Bernal-Vicente A., Diaz-Vivancos P., Sanchez-Blanco M., Hernandez J. (2017). Plant responses to salt stress: Adaptive mechanisms. Agronomy.

[31] Franco J.A., Fernández J.A., Bañón S., González A. (1997). Relationship between the effects of salinity on seedling leaf area and fruit yield of six muskmelons cultivars. J. Hortic. Sci..

[32] Rodríguez P., Torrecillas A., Morales M.A., Ortuño M.F., Sánchez-Blanco M.J. (2005). Effects of NaCl salinity and water stress on growth and leaf water relations of *Asteriscus maritimus* plants. Environ. Exp. Bot..

[33] Muchate N.S., Rajurkar N.S., Suprasanna P., Nikam T.D. (2019). NaCl induced salt adaptive changes and enhanced accumulation of 20-hydroxyecdysone in the *in vitro* shoot cultures of *Spinacia oleracea* (L.). Sci. Rep..

[34] Haider M.S., Barnes J.D., Cushman J.C., Borland A.M. (2012). A CAM- and starch-deficient mutant of the facultative CAM species *Mesembryanthemum crystallinum* reconciles sink demands by repartitioning carbon during acclimation to salinity. J. Exp. Bot..

[35] Matsuoka T.A., Onozawa K., Sonoike S., Kore-eda S. (2018). Crassulacean acid metabolism induction in *Mesembryanthemum crystallinum* can be estimated by non-photochemical quenching upon actinic illumination during the dark period. Plant Cell Physiol..

[36] Broettoa F., Duarteb H.M., Lüttge U. (2007). Responses of chlorophyll fluorescence parameters of the facultative halophyte and C_3_–CAM intermediate species *Mesembryanthemum crystallinum* to salinity and high irradiance stress. J. Plant Physiol..

[37] Stepien P., Johnson G.N. (2009). Contrasting responses of photosynthesis to salt stress in the glycophyte Arabisopsis and the halophyte Thellungiella: Role of plastid terminal oxidase as an alternative election sink. Plant Physiol..

[38] Shin Y.K., Bhandari S.R., Jo J.S., Song J.W., Cho M.C., Yang E.Y., Lee J.G. (2020). Response to Salt Stress in Lettuce: Changes in Chlorophyll Fluorescence Parameters, Phytochemical Contents, and Antioxidant Activities. Agronomy.

[39] Wang X., Mao Z., Zhang J., Hemat M., Huang M., Cai J., Qin Z., Dai T., Dong J. (2019). Osmolyte accumulation plays important roles in the drought priming induced tolerance to post-anthesis drought stress in winter wheat (*Triticum aestivum* L.). Environ. Exp. Bot..

[40] Santos C.V. (2004). Regulation of chlorophyll biosynthesis and degradation by salt stress in sunflower leaves. Sci. Hortic..

[41] Guan Q., Tan B., Kelley T.M., Tian J., Chen S. (2020). Physiological changes in *Mesembryanthemum crystallinum* during the C_3_ to CAM transition induced by salt stress. Front. Plant Sci..

[42] Hsouna A.B., Ghneim-Herrera T., Romdhane W.B., Dabbous A., Saad R.B., Brini F., Abdelly C., Hamed B.K. (2020). Early effects of salt stress on the physiological and oxidative status of the halophyte *Lobularia maritima*. Funct. Plant. Biol..

[43] Blum A., Munns R., Passioura J.B., Turner N.C., Sharp R.E., Boyer J.S., Nguyen H.T., Hsiao T.C., Verma D.P.S., Hong Z. (1996). Genetically engineered plants resistant to soil drying and salt stress: How to interpret osmotic relations?. Plant Physiol..

[44] Abogadallah G.M. (2010). Antioxidative defense under salt stress. Plant Signal. Behav..

[45] Hunt R., Causton D.R., Shipley B., Askew A.P. (2002). A modern tool for classical plant growth analysis. Ann. Bot..

[46] Agarie S., Shimoda T., Shimizu Y., Baumann K., Sunagawa H., Kondo A., Ueno O., Nakahara T., Nose A., Cushman J.C. (2007). Salt tolerance, salt accumulation, and ionic homeostasis in an epidermal bladder-cell-less mutant of the common ice plant *Mesembryanthemum crystallinum*. J. Exp. Bot..

[47] Garnier E., Shipley B., Roumet C., Laurent G. (2001). A standardized protocol for the determination of specific leaf area and leaf dry matter content. Funct. Ecol..

[48] He J., Tan B.H.G., Qin L. (2011). Source-to-sink relationship between green leaves and green pseudobulbs of C3 orchid in regulation of photosynthesis. Photosynthetica.

[49] He J., Teo L.C.D. (2007). Susceptibility of CAM *Dendrobium* Burana Jade green leaves and green flower petals to high light under tropical natural conditions. Photosynthetica.

[50] Welburn A.R. (1994). The spectral determination of chlorophylls a and b, as well as carotenoids, using various solvents with spectrophotometers of different resolution. J. Plant Physiol..

[51] Bates L.S., Waldren R.P., Teare I.D. (1973). Rapid determination of free proline for water- stress studies. Plant Soil.

[52] Dubois M., Gilles K.A., Hamilton J.K., Rebers A., Smith F. (1956). Colorimetric method for determination of sugars and related substances. Anal. Chem..

[53] Leipner J., Fracheboud Y., Stamp P. (1997). Acclimation by suboptimal temperature diminishes photooxidative damage in maize leaves. Plant. Cell Environ..

[54] Ragee S., Abdel-Aal E.M., Noaman M. (2006). Antioxidant activity and nutrient composition of selected cereals for food use. Food Chem..

